# Analysis of the main causes of chicken carcass condemnation in slaughterhouses in Mato Grosso, Brazil

**DOI:** 10.29374/2527-2179.bjvm007526

**Published:** 2026-09-14

**Authors:** Michael Doughlaus Krindges, Amanda Tavares da Mata, Tony Edgley Catão Tenório, Yuri Duarte Porto, Vinicius Silva Castro, Adelino da Cunha, Ricardo César Tavares Carvalho

**Affiliations:** 1 Programa de Pós-Graduação em Biociência Animal, Universidade de Cuiabá, Cuiabá, MT, Brazil.; 2 Universidade de Cuiabá, Cuiabá, MT, Brazil.; 3 Programa de Pós-Graduação em Nutrição, Alimentos e Metabolismo, Faculdade de Nutrição, Universidade Federal de Mato Grosso, Cuiabá, MT, Brazil.; 4 University of Lethbridge, Lethbridge, Alberta, Canada.

**Keywords:** slaughter, poultry farming, midwestern Brazil, sanitary inspection, animal production, abate, avicultura, centro-oeste, inspeção sanitária, produção animal

## Abstract

Pre-slaughter inspection activities encompass clinical, documentary, and sanitary checks; post-mortem inspection identifies abnormalities in chicken carcasses, such as lesions caused by disease or inadequate handling and processing, leading to partial or total condemnation. The objectives were to determine the causes of condemnation for poultry carcasses slaughtered in Mato Grosso, identify the triggering factors, and evaluate the economic impact on the chicken production chain between January 2017 and December 2024. This was a retrospective, exploratory study using data from the Federal Inspection Service's Management Information System (SIGSIF) and reports from inspected establishments. Of the 1,251,623,202 chickens slaughtered, 9.5% (118,990,903) of the carcasses were condemned: 2.69% (33,759,843) totally and 6.8% (85,231,060) partially. Gastrointestinal contamination showed the highest percentages for both totally and partially condemned carcasses (57.16% and 37.55%, respectively), followed by cellulitis, accounting for 18% of both types of condemnation. Repugnant appearance (7.94%) and bruising/fractures (11.29%) ranked third among total and partial condemnations. Factors influencing condemnations included environmental conditions (temperature and relative humidity) as well as handling practices during rearing, transport, and processing. Economic losses associated with the 118,990,903 condemned birds during this period totaled R$ 1,365,420,611.93. We concluded that the partial and total condemnation of poultry involves a complex set of factors, ranging from on-farm management to processing. Understanding the situation in the state enables the formulation of measures to prevent and mitigate condemnations and the resulting economic losses associated with the disposal of these birds.

## Resumo

As atividades de inspeção pré-abate abrangem verificações clínicas, documentais e sanitárias. A inspeção *post-mortem* identifica anomalias em carcaças de frango, como lesões causadas por doenças ou falhas no manejo e processamento, levando à condenação parcial ou total. O objetivo foi determinar as causas de condenação de carcaças de aves abatidas em Mato Grosso, identificar os fatores desencadeantes e avaliar o impacto econômico na cadeia produtiva entre janeiro de 2017 e dezembro de 2024. Tratou-se de um estudo retrospectivo e exploratório, utilizando dados do Sistema de Informações Gerenciais do Serviço de Inspeção Federal (SIGSIF) e relatórios de estabelecimentos inspecionados. Do total de 1.251.623.202 frangos abatidos, 9,5% (118.990.903) das carcaças foram condenadas: 2,69% (33.759.843) totalmente e 6,8% (85.231.060) parcialmente. A contaminação gastrointestinal apresentou os maiores percentuais nas condenações totais e parciais (57,16% e 37,55%, respectivamente); a celulite representou 18% em ambas, ocupando o segundo lugar em frequência. Aspecto repugnante (7,94%) e contusões/fraturas (11,29%) ocuparam a terceira posição nas condenações totais e parciais. Os fatores que influenciaram as condenações incluíram condições ambientais (temperatura e umidade relativa) e práticas de manejo durante a criação, transporte e processamento. As perdas econômicas totalizaram R$ 1.365.420.611,93, associadas às 118.990.903 aves condenadas. Conclui-se que a condenação parcial e total de aves envolve fatores desde o manejo na granja até o processamento. Compreender a situação permite a formular medidas para prevenir e mitigar as condenações e as perdas econômicas associadas ao descarte dessas aves.

## Introduction

Brazil produced 14.972 million tons of chicken meat in 2023, of which 5.295 million tons were exported, ranking Brazil as the second-largest chicken producer and the world's leading exporter (Associação Brasileira de Proteína Animal, 2025). The chicken meat production process must adhere to strict production standards, with industrial inspection being one of the mechanisms adopted to ensure food safety and sustain this production. Carcasses with any type of abnormality indicative of an animal health problem that may pose a risk to consumers are considered unfit for human consumption and may be partially or totally condemned (Souza et al., 2019).

The grounds for condemnation are divided into pathological causes, arising from disease-associated lesions, and technopathies, resulting from defects in the slaughterhouse during catching, loading, transport, hanging, or the use of equipment and utensils in the slaughterhouse. Condemnation due to pathological or operational technological defects can be classified as partial or total depending on the degree of injury, in accordance with criteria established by the Federal Inspection System (SIF) (Souza et al., 2016). The main causes of condemnation (partial and total) determined by the SIF in accordance with Ordinance 210/98 are due to diseases such as abscess, airsacculitis, arthritis, cachexia, cellulitis, colibacillosis, dermatosis, edema, myopathy, neoplasia, salpingitis, septicemia, ascitic syndrome and hemorrhagic syndrome, and technological failures such as repugnant appearance, inadequate bleeding, gastrointestinal and biliary contamination, contusion, excessive scalding and delayed evisceration (Brasil, 1998, 2017).

Notable causes for the condemnation of poultry carcasses across various countries include cachexia, ascites, arthritis, septicemia, and death during transport. These were identified through quality control measures implemented by animal product inspection services in countries such as Ethiopia (Abdi et al., 2023), Iran (Azizpour & Amirajam, 2024), Norway (Gretarsson et al., 2023), the United States (Herenda & Jakel, 1994), France (Lupo et al., 2010), Thailand (Klaharn et al., 2022; Pirompud et al., 2023), Portugal (Saraiva et al., 2024), Denmark (Alfifi et al., 2020), and Germany (Schulze Bernd et al., 2020).

In Brazil, gastrointestinal contamination, bruising/fractures, and repulsive appearance are the most frequent causes for the condemnation of chicken carcasses, followed by cellulitis, ascites, and arthritis. These issues have been identified through sanitary surveillance at both the national (Oliveira et al., 2016) and the state levels, with data from states such as Goiás (Mendonça et al., 2022; Santana et al., 2008), Bahia (Barreto et al., 2022), Espírito Santo (Candido et al., 2021), Rio Grande do Sul (Ferreira et al., 2012; Jacobsen & Flôres, 2008), Paraná (Goscinscki, 2016; Kanabata et al., 2023; Pissolitto & Piassa, 2023;), and Minas Gerais (Moura et al., 2012), as well as at the municipal level, with records from the cities of Belo Horizonte, MG (Matos et al., 2023) and Manaus, AM (Monteiro et al., 2022). These condemnations result in the disposal of whole birds or parts thereof. Since chickens produced in Brazil are marketed both domestically and internationally, this results in economic losses for the chicken production chain (Souza et al., 2019).

In Brazil, the Ministry of Agriculture and Livestock (MAPA - *Ministério da Agricultura Pecuária e Abastecimento*) manages herds of slaughtered animals through the Federal Inspection Service Management Information System (SIGSIF - *Sistema de Informações Gerenciais do Serviço de Inspeção Federal*), created in 2003, through records of condemnations diagnosed by the Federal Inspection Service (SIF *- Serviço de Inspeção Federal*) during slaughter (Oliveira et al., 2016). This activity is carried out by the veterinarian (SIF Auditor) who, based on Ordinance No. 210 of November 10, 1998 (Brasil, 1998; Oliveira et al., 2016), assesses occurrences during the poultry slaughter procedure, according to the Regulation of Industrial and Sanitary Inspection of Products of Animal Origin (RIISPOA-*Regulamento da Inspeção Industrial e Sanitária de Produtos de Origem Animal*) (Brasil, 2017). SIGSIF records are a source of information on animal condemnations, serving as a basis for epidemiological calculations to assess sanitary conditions in all slaughters carried out (Oliveira et al., 2016; Silva, 2017).

The aims of this study were to determine the primary causes for the total and/or partial condemnation of poultry carcasses at slaughterhouses in the state of Mato Grosso, identify the factors triggering these condemnations by reviewing information from scientific studies, and assess the economic impact of these condemnations on the broiler chicken supply chain during the study period.

## Material and methods

A retrospective study of data available in the Federal Inspection Service Management Information System (SIGSIF) was carried out, based on reports generated in establishments under the Federal Inspection Service from January 2017 to December 2024 (Brasil, 2023). The poultry slaughter reports were analyzed using the year/month and Federal Unit (UF) filters related to the number of birds slaughtered per year in the state, as well as the number of birds slaughtered in the state of Mato Grosso during this period. The variables evaluated were: quantity condemned”, “causes for the condemnation of poultry”, “UF”, “month”, “year”, “destination of condemnation”, and “number of birds slaughtered”.

Any inconsistencies, such as condemnations due to diseases not compatible with the species being analyzed, were manually removed prior to data analysist, using the "causes for the condemnation of poultry" variable as a reference.

Annual economic losses were calculated using the following equation, adapted to Souza et al. (2019):


$$Annual\;economic\;losses\;=\;number\;of\;condemned\;carcasses\;per\;year\;\times \;average\;meat\;yield\;per\;carcass\;\left(2.5\;kilogram\right)\;\times \;average\;annual\;price\;per\;kilo\;\left(R\$\;4.59\right)$$ (1)

The records were grouped into tables using Microsoft Excel. Descriptive statistical analysis was used to calculate the frequencies of total and partial condemnations by cause. Additionally, the variation in the frequency of condemnations over the years and their distribution across months for the main causes of total and partial condemnations were investigated using regression analysis. Seasonality analysis was performed using the Friedman test. The analyses were performed using R 4.1.0 software (R Core Team, 2021).

## Results

**Figure 1:**
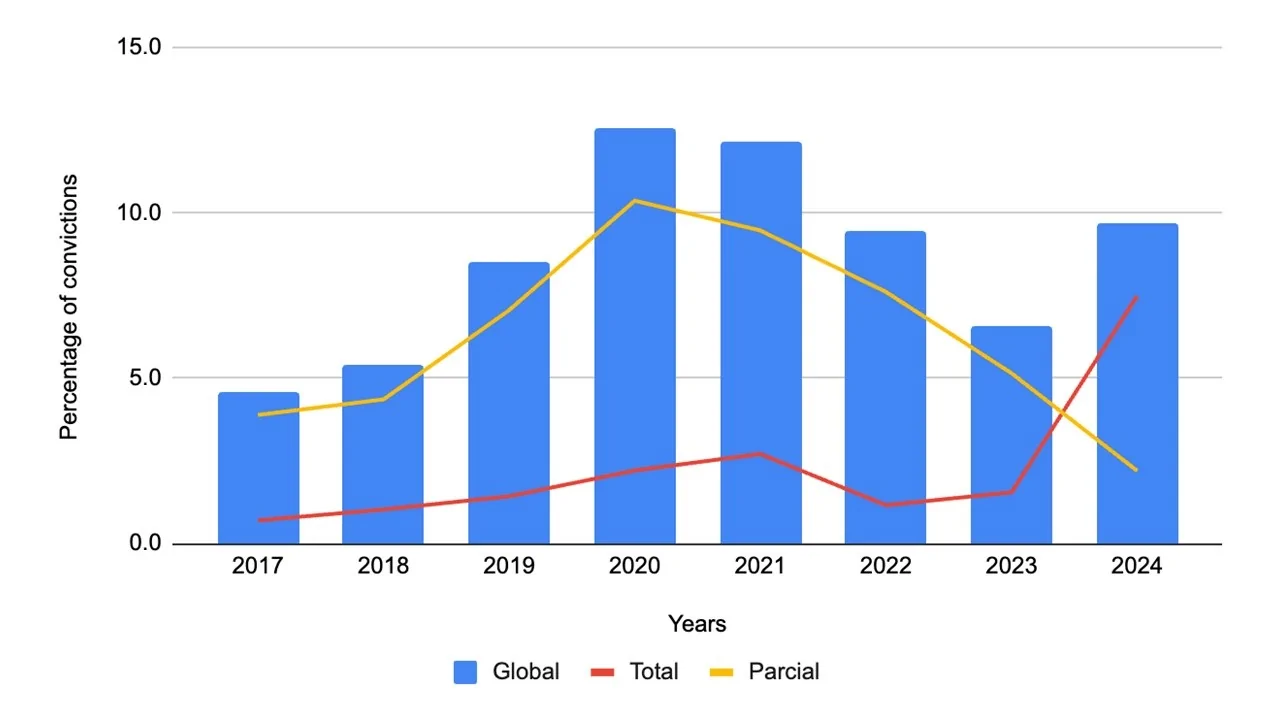
Percentage of overall, total, and partial condemnations relative to the total number of chickens slaughtered (*n* = 1,251,623,202) in poultry slaughterhouses inspected by the Federal Inspection Service (SIF) in the state of Mato Grosso, Brazil, from January 2017 to December 2024.

According to data stored by the Ministry of Agriculture and Livestock, from January 2017 to December 2024, a total of 1,251,623,202 chickens were slaughtered in slaughterhouses located in the state of Mato Grosso, Brazil. Of these carcasses (slaughtered chickens), 9.5% (118,990,903) were condemned, of which 2.69% (33,759,843) were fully condemned. However, 6.8% (85,231,060) of these carcasses had a partial condemnation (Figure 1), revealing the epidemiology of chicken carcass condemnation over the eight-year period (2017-2024).

When evaluating the distribution of the average percentages of overall condemnation of poultry carcasses in Mato Grosso, the highest overall condemnation (combined total and partial condemnations) percentages were observed in 2020 and 2021 at 12.58% and 12.17%, respectively. Partial condemnations were the largest contributors, with percentages of 10.35% and 9.47% in these 2 years (Figure 1).

**Figure 2:**
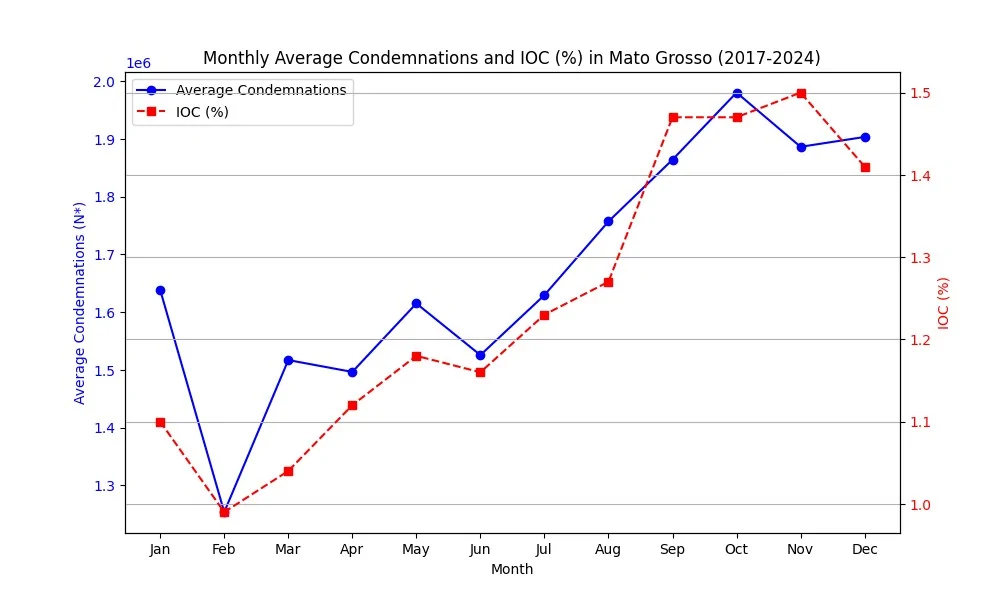
Average monthly number of condemnations and Condemnation Occurrence Index (COI) for poultry slaughterhouses inspected by the Federal Inspection Service (SIF) in the state of Mato Grosso,Brazil, from January 2017 to December 2024 (*n* = 1,251,623,202).

When the average monthly condemnations over the 8 years were evaluated, the condemnation occurrence index (COI), representing the ratio of the average number of condemned carcasses to the average total number of animals slaughtered in the period between 2017 and 2024, increased from September to December, ranging from 1.0% to 1.5% (Figure 2).

**Figure 3:**
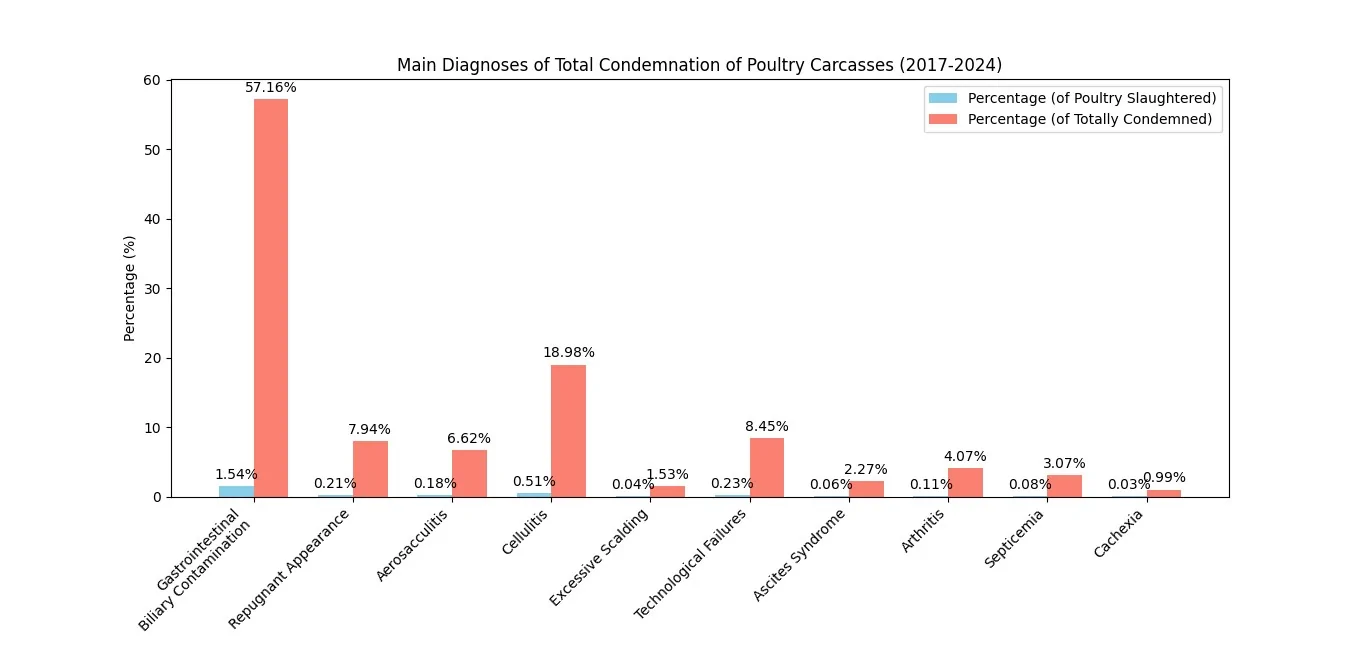
Percentage of the main causes of total condemnation of poultry carcasses in the state of Mato Grosso, Brazil, from January 2017 to December 2024, relative to the total number of birds slaughtered (n = 1,251,623,202) and to the total number of completely condemned carcasses (n = 33,759,843).

Among the ten main diagnoses contributing to the total condemnation of chicken carcasses, gastrointestinal and biliary contamination accounted for the largest proportion, representing 1.54% of the 1,251,623,202 birds slaughtered in Mato Grosso from 2017 to 2024 (Figure 3). This was followed by the diagnosis of cellulitis (0.51%), technological failures (0.23%), and repugnant appearance (0.21%) (Figure 3).

**Figure 4:**
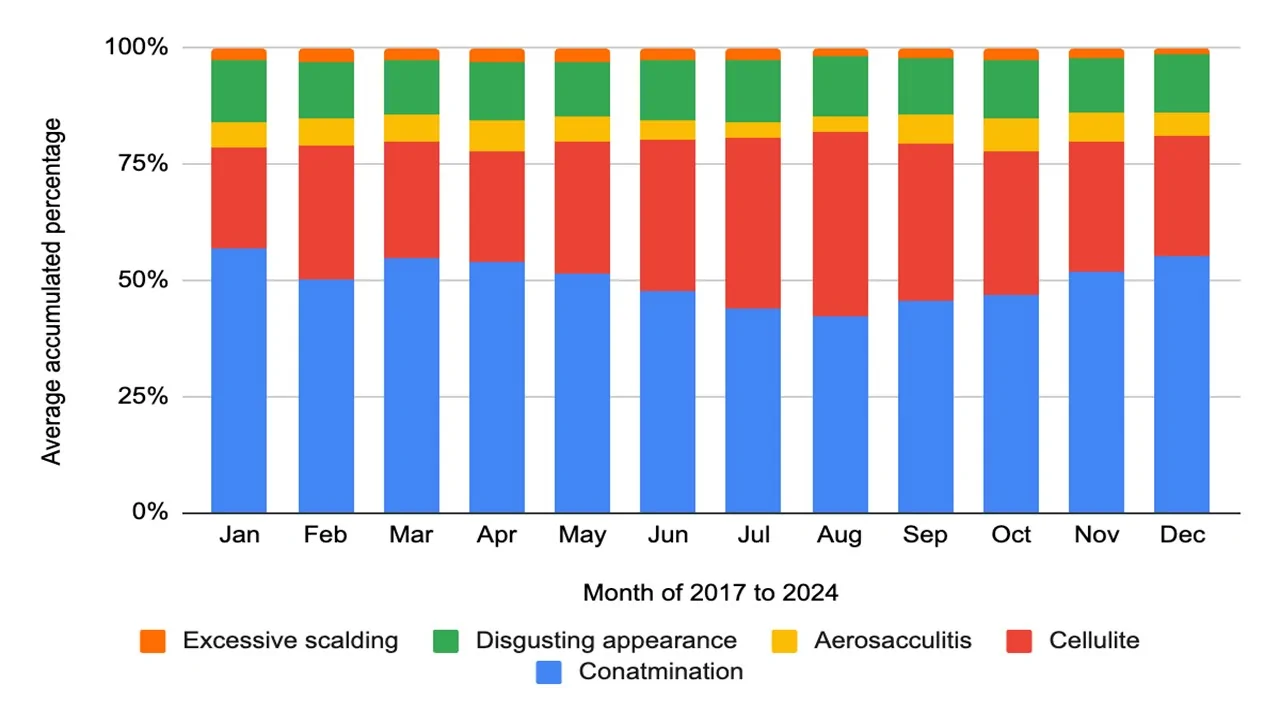
Average monthly distribution (%) of the main causes of total carcass condemnation recorded by the Federal Inspection Service in poultry slaughterhouses in Mato Grosso, Brazil, from January 2017 to December 2024.

An analysis of the causes contributing to total carcass condemnation of 33,759,843 carcasses between 2017 and 2024 reveals that the primary causes, in descending order, were gastrointestinal and biliary contamination (57.16%), cellulitis (18.98%), processing defects (8.45%), and repulsive appearance (7.94%) (Figure 3; data represent average annual percentages). An assessment of monthly contributions from January 2017 to December 2024 shows that gastrointestinal and biliary contamination accounted for 50% or more of total condemnations from January to May and in November and December (Figure 4). Cellulitis also contributed significantly to total carcass condemnation from June to October (Figure 3).

**Figure 5:**
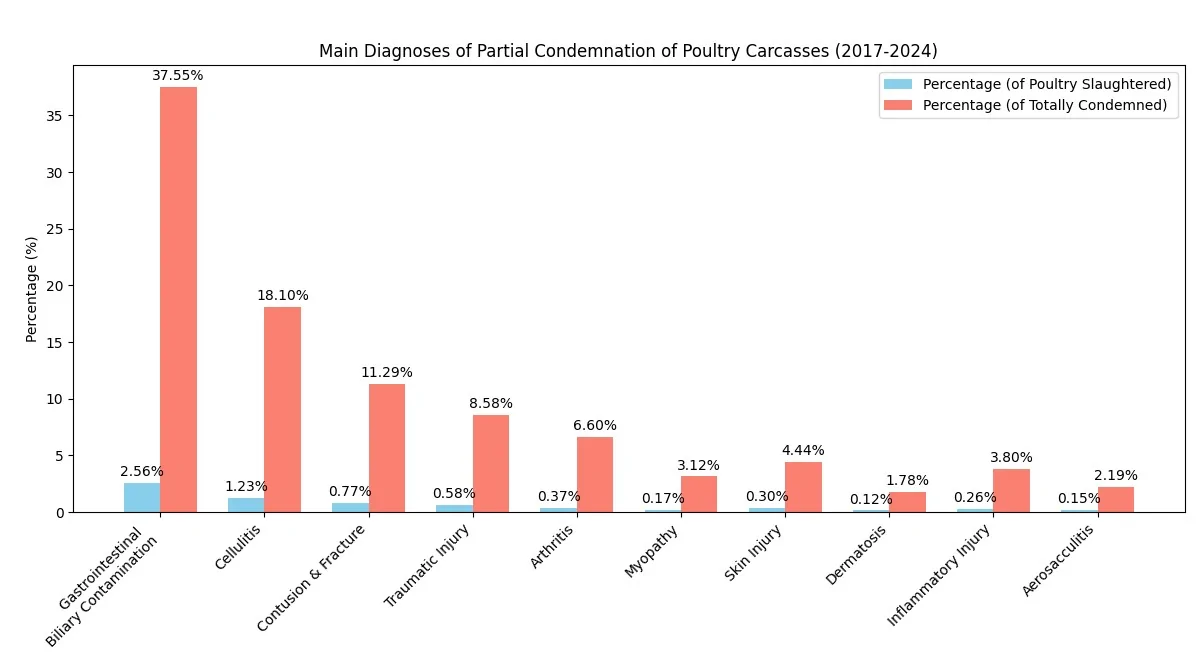
Percentage of the main causes of partial condemnation of poultry carcasses in the state of Mato Grosso, Brazil, from January 2017 to December 2024, relative to the total number of birds slaughtered (n = 1,251,623,202) and to the total number of partially condemned carcasses (n = 85,231,060).

Ten diagnoses contributed to partial condemnation of chicken carcasses, among which gastrointestinal and biliary contamination represented 2.56% of the total (1,251,623,202 carcasses) birds slaughtered in Mato Grosso, from 2017 to 2024 (Figure 5). Other causes included cellulitis (1.23%), contusion and fracture (0.77%), traumatic injury (0.58%), and arthritis (0.37%) (Figure 4). The last three diagnoses differ from those that occurred in the total condemnation of chicken carcasses.

**Figure 6:**
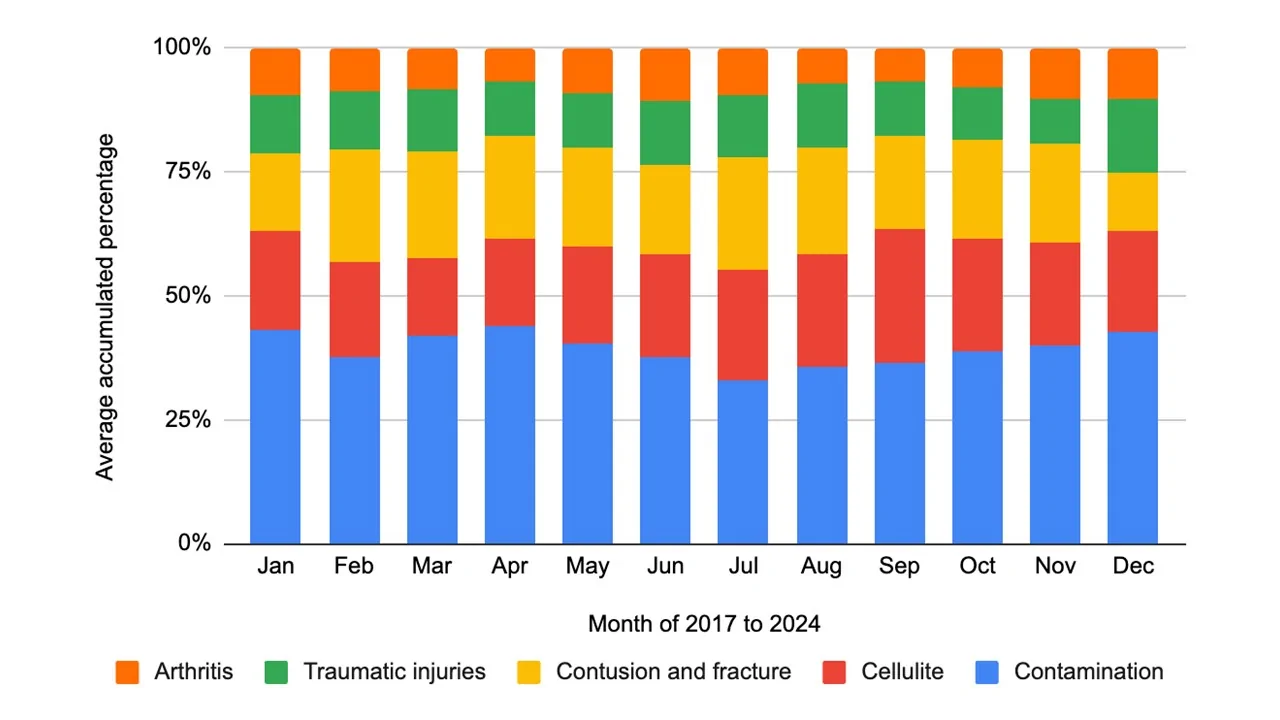
Average monthly distribution (%) of the main causes of partial carcass condemnation recorded by the Federal Inspection Service in poultry slaughterhouses in Mato Grosso, Brazil, from January 2017 to December 2024.

Gastrointestinal and biliary contamination (accounting for 37.55% of these cases), cellulitis (18.10%), bruising and fractures (11.29%), traumatic injury (8.58%), and arthritis (6.60%) were the leading issues resulting in the 85,231,060 chicken carcasses (6.8% of 1,251,623,202 carcasses) subjected to partial condemnation during the period (2017-2024), (Figure 5). An analysis of the average monthly percentages of the issues contributing to partial carcass condemnation during the evaluated period revealed that gastrointestinal and biliary contamination had the greatest influence; however, the detected percentages remained below 40%, particularly between June and October (Figure 6).

Importantly, between January 2017 and December 2024, gastrointestinal and biliary contamination and cellulitis were the most frequently diagnosed causes of total and partial chicken carcass condemnation by year or month in terms of both frequency and percentage. Gastrointestinal and biliary contamination, a processing-related defect, is the primary contributing factor to both total and partial chicken carcass condemnation in the state of Mato Grosso. Among other processing-related defects, objectionable appearance and over-scalding contributed to total carcass condemnation (Figure 4) during this period, whereas traumatic injuries, contusions, and fractures contributed to partial condemnation (Figure 6).

**Figure 7:**
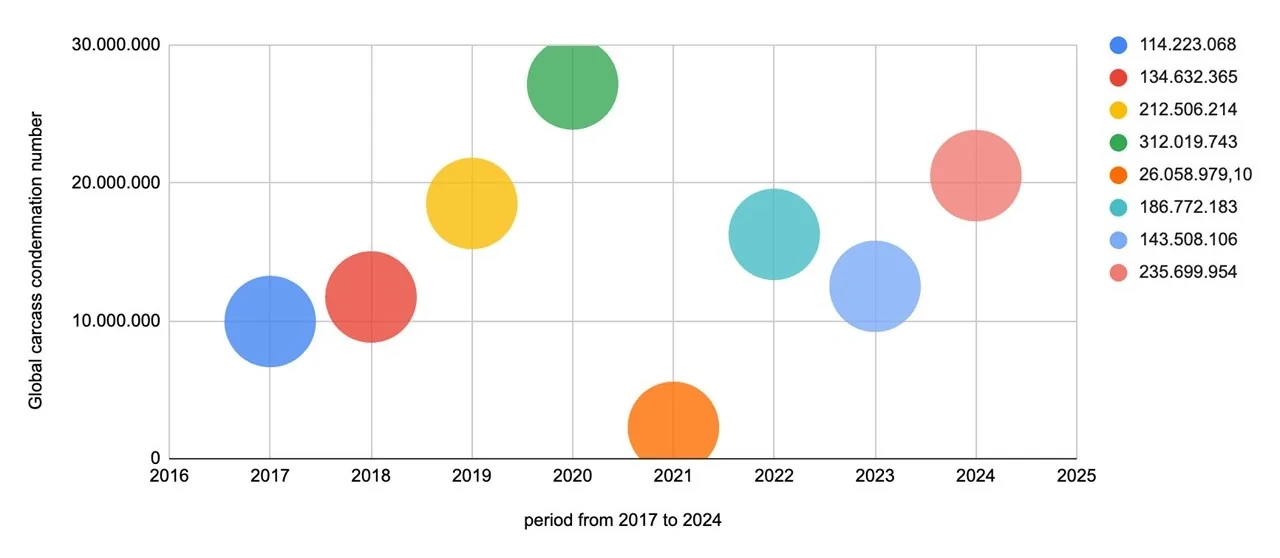
Annual number of chickens condemned (total and partial condemnations) by the Federal Inspection Service in poultry slaughterhouses in Mato Grosso, Brazil, from January 2017 to December 2024, and the corresponding estimated annual economic losses.

The production cost of live chickens in Paraná in January 2024 was R$ 4.59, according to the Brazilian Agricultural Research Corporation (Empresa Brasileira de Pesquisa Agropecuária, 2026). This figure was used as a reference to calculate the financial losses resulting from these condemnations, given that the state is the country's largest producer of broiler chickens. Assuming an average slaughter weight of approximately 2.5 kg, the financial loss associated with condemnation (the sum of total and partial condemnations) of 118,990,903 birds during the study period (2017-2024) amounted to R$ 1,365,420,611.93 (approximately R$ 1,37 billion) (Figure 7). These losses are substantial, considering the scale of the poultry supply chain and its production costs, which encompass everything from feed, facilities, energy, water, and labor to transportation and slaughter.

It is worth noting that the years 2020 and 2024 recorded the highest numbers of overall condemnations, with 27,191,263 and 20,540,301 birds condemned (total and partial), resulting in losses of R$ 312,019,742.90 and R$ 235,699,954.00, respectively, as illustrated in Figure 7.

## Discussion

The quality of chicken meat in industrial production can be affected by various diseases and pathological changes. These changes can result in partial destruction of organs or the entire carcass, causing economic losses to the industry and the country (Abdi et al., 2023).

The most common pathological causes for condemnation observed in this study, affecting carcasses condemned either wholly or partially, were cellulitis, airsacculitis, and arthritis. Additionally, conditions such as septicemia, ascites syndrome, and cachexia were present in wholly condemned carcasses, whereas dermatoses, myopathy, and skin inflammation were observed in partially condemned carcasses. The predominant issue regarding processing-related defects in wholly and/or partially condemned carcasses was gastrointestinal and biliary contamination. Repulsive appearance and over-scalding occurred in wholly condemned carcasses, while processing-related causes for partial condemnation included traumatic lesions, bruising, and fractures (Brasil, 2017; Souza et al., 2016).

The two main causes of poultry carcass condemnation showing the highest incidence rates were gastrointestinal/ biliary contamination and cellulitis, for both total and partial condemnation. The percentages for cellulitis were similar: 18.10% for total and 18.98% for partial condemnation.

Gastrointestinal and biliary contamination of carcasses in slaughterhouses is a recurring problem, primarily caused by failures during automated evisceration. This stage is a contributing factor, as perforation of the digestive tract can occur, contaminating carcasses with gastrointestinal and biliary contents (Mendonça et al., 2022; Procópio, 2020). One contributing factor is inadequate fasting of the birds. While a fasting period of 8-12 hours is crucial, its duration can be affected by company logistics and waiting times at the slaughterhouse. Emptying the intestines and crop at the time of slaughter and evisceration is essential to reduce contamination (Mendonça et al., 2022; Procópio, 2020). Furthermore, bird-to-bird variation resulting from weight differences interferes with the adjustment of evisceration machinery, leading to gastrointestinal tract perforations and carcass contamination (Mendonça et al., 2022; Procópio, 2020). Such adjustments to the machinery are not frequently performed in processing facilities (Santana et al., 2008).

Traumatic injuries, bruises, and fractures, as well as skin conditions such as cellulitis and other dermatoses (which can result in a repulsive appearance), may arise due to factors such as flaws in pre-slaughter management within the poultry house, high stocking density, poor litter quality, and inadequate nutrition (Santos et al., 2019). A maximum density of 15 birds per m^2^ is recommended to prevent such injuries (Santana et al., 2008). Other contributing factors may be related to the stages of catching, loading, transport, receiving, and the handling involved in removing birds from cages and hanging them on the conveyor line (Almeida et al., 2017).

Another technical defect is excessive scalding, a factor that contributed to the total condemnation of chicken carcasses between 2017 and 2024. This is one of the causes of condemnation associated with failures during poultry slaughter and processing (Souza et al., 2019). Excessive scalding can result from processing line stoppages, high water temperatures, and improperly adjusted equipment. Consequently, the carcasses exhibit a dried-out muscle texture or a cooked-meat appearance, as well as a whitish discoloration on the lower breast (Souza et al., 2019).

Airsacculitis and ascitic syndrome (pulmonary hypertension syndrome) are common and serious respiratory diseases in birds, with ascitic being the leading cause of death (Souza et al., 2021; Spanamberg et al., 2013). Airsacculitis manifests with lymphoid hyperplasia, pneumonia, bronchitis, and bronchopneumonia, and is associated with bacterial, viral, and, secondarily, fungal agents (Spanamberg et al., 2013). Ascitic syndrome, in turn, is a multifactorial problem influenced by the environment and genetics, and is characterized by hypoxemia, cardiorespiratory system overload, fluid accumulation, and right ventricular hypertrophy (Souza et al., 2021).

Intensive poultry production in tropical and subtropical regions is influenced by environmental factors, such as temperature and relative humidity, that primarily affect production, transport, and processing at the slaughterhouse (Lopes et al., 2026; Vieira et al., 2010). A comfortable environment for adult birds is characterized by temperatures between 16 °C and 23 °C and relative humidity levels of 50% to 70% (Braz et al., 2024; Oliveira et al., 2006).

The state of Mato Grosso lies entirely within the planet's tropical zone, resulting in a hot climate characterized by a rainy season in summer (November-April) and a dry season in winter (May-October). Mato Grosso encompasses three biomes: the Amazon, the Cerrado, and the Pantanal (Curado et al., 2024; Melo et al., 2025; Soria et al., 2024). Across its three biomes, the state recorded average monthly temperatures of 24.20 °C in June and 27.27 °C in September between 2013 and 2024, while the average relative humidity was 62.64% in August and 87.33% in February (Supplementary Figure 1) (Melo et al., 2025; Curado et al., 2024; Soria et al., 2024).

As shown in Figures 4 and 6, gastrointestinal and biliary contamination was more prominent between November and April; it accounted for more than 50% of total condemnations and approximately 40% of partial condemnations during this period (Figures 5 and 6). During these months, the average temperature ranged from 26.24 °C to 26.06 °C, and relative humidity ranged from 82.59% to 84.94% (December-April) (Supplementary Figure 1).

Regarding the total condemnation of poultry carcasses, cellulitis was notably prevalent from June to October, a trend not observed in cases of partial condemnation, a period during which average temperatures ranged from 24.20 °C (June) to 27.27 °C (September) and relative humidity ranged from 62.64% (August) to 67.29% (June) (Supplementary Figure 1); contamination was more frequent during the rainy season. Cellulitis was the second leading cause of total carcass condemnation during the dry season (June to October). Broiler production is carried out in a closed system designed to control the birds' environment via climate control systems; nevertheless, seasonal changes in the health and welfare of these animals are observed (Apalowo et al., 2024; Braz et al., 2024; Kuo et al., 2025; Lin et al., 2005; Part et al., 2016).

Certain specific pathologies, such as airsacculitis, ascites, arthritis, and cellulitis, is predominant due to the actual influence of seasonal stressors (temperature and relative humidity) in tropical and subtropical climates. Airsacculitis is related to mean, maximum, and minimum temperatures; ascites is associated with winter conditions (dry weather and low temperatures); and arthritis and cellulitis respond to significant fluctuations and high levels of environmental humidity (Lopes et al., 2026).

In this study, cellulitis accounted for approximately 18% of partial or total poultry carcass condemnations. According to Lopes et al. (2026), skin lesions in broiler chickens show a negative correlation with temperature range and a positive correlation with humidity. Furthermore, high stocking density combined with humidity exacerbates contact dermatitis (Lopes et al., 2026). It is hypothesized that heavy rainfall leaves the litter damp and/or causes the birds to huddle together, thereby increasing ammonia levels and facilitating the entry of pathogens (such as *Mycoplasma* spp. and *Escherichia coli*) into joints and tissues (Lopes et al., 2026).

Gastrointestinal and biliary contamination is attributed to weight variations that interfere with the adjustment of evisceration machinery, leading to perforation of the gastrointestinal tract (Mendonça et al., 2022; Procópio, 2020; Santana et al., 2008). Air temperature and relative humidity can influence bird weight, as higher ambient temperatures reduce voluntary feed intake in broiler chickens (Oliveira et al., 2006). High relative humidity (>60%) negatively affects feed intake; therefore, both factors impact weight gain in the birds (Oliveira et al., 2006).

Studies conducted in other Brazilian states have described the causes of total and partial chicken carcass condemnations. Pathological conditions, such as cellulitis causing condemnations in Bahia (Barreto et al., 2022), Espírito Santo (Candido et al., 2021; Dias et al., 2017), and Paraná (Jaguezeski et al., 2020), as well as ascites syndrome in Minas Gerais (Matos et al., 2023), were the primary issues. Regarding processing-related defects, the main issues identified were bruising in Bahia (Barreto et al., 2022); bruising and fractures in Espírito Santo (Candido et al., 2021; Dias et al., 2017); gastrointestinal and biliary contamination in Rio Grande do Sul (Ferreira et al., 2012) and Paraná (Jaguezeski et al., 2020); "repugnant appearance" in Rio Grande do Sul (Ferreira et al., 2012) and Minas Gerais (Matos et al., 2023); and cachexia in Rio Grande do Sul (Ferreira et al., 2012).

Carcass condemnations represent food waste (protein) and financial losses (Lopes et al., 2026). Total and partial condemnations contribute to economic losses in the chicken production chain (Mendes et al., 2024; Souza et al., 2019). An economic loss of R$ 1,365,420,611.93, associated with 118,990,903 condemned birds, was observed during the study period (2017-2024) (Figure 7). The loss resulting from carcass condemnations highlights the importance of investigating the factors that trigger condemnation, to minimize economic losses in the poultry sector (Mendes et al., 2024).

## Conclusion

We concluded that the primary causes of post-mortem condemnation of poultry slaughtered in facilities supervised by the Federal Inspection Service in the state of Mato Grosso between 2017 and 2024 were gastrointestinal and biliary contamination, accounting for 57.16% of total and 37.55% of partial condemnations. This was followed by cellulitis, representing approximately 18% in both categories. Repugnant appearance ranked third for total condemnations (7.94%), whereas bruising and fractures ranked third for partial condemnations (11.29%). Factors associated with these condemnations include pre-slaughter stages, encompassing rearing, catching, and transport, as well as processing conditions and environmental factors such as temperature and relative humidity. Identifying these factors may support interventions aimed at minimizing the economic impact on the production chain.

## Supplementary Material

Supplementary material accompanies this paper.

Supplementary Figure 1 Average monthly temperature (°C) and relative humidity (%) in the state of Mato Grosso, Brazil, based on data from 2013 to 2024.

This material is available as part of the online article from https://doi.org/10.29374/2527-2179.bjvm007526
