# Supplementary material for: Analysis of the main causes of chicken carcass condemnation in slaughterhouses in Mato Grosso, Brazil
Source: Braz J Vet Med. 2026 Sep 14;48:e007526. doi: 10.29374/2527-2179.bjvm007526 (PMC13577607; doi:10.29374/2527-2179.bjvm007526)
Supplement: Supplementary file 1 [file bjvm-48-e007526-suppl01.pdf]

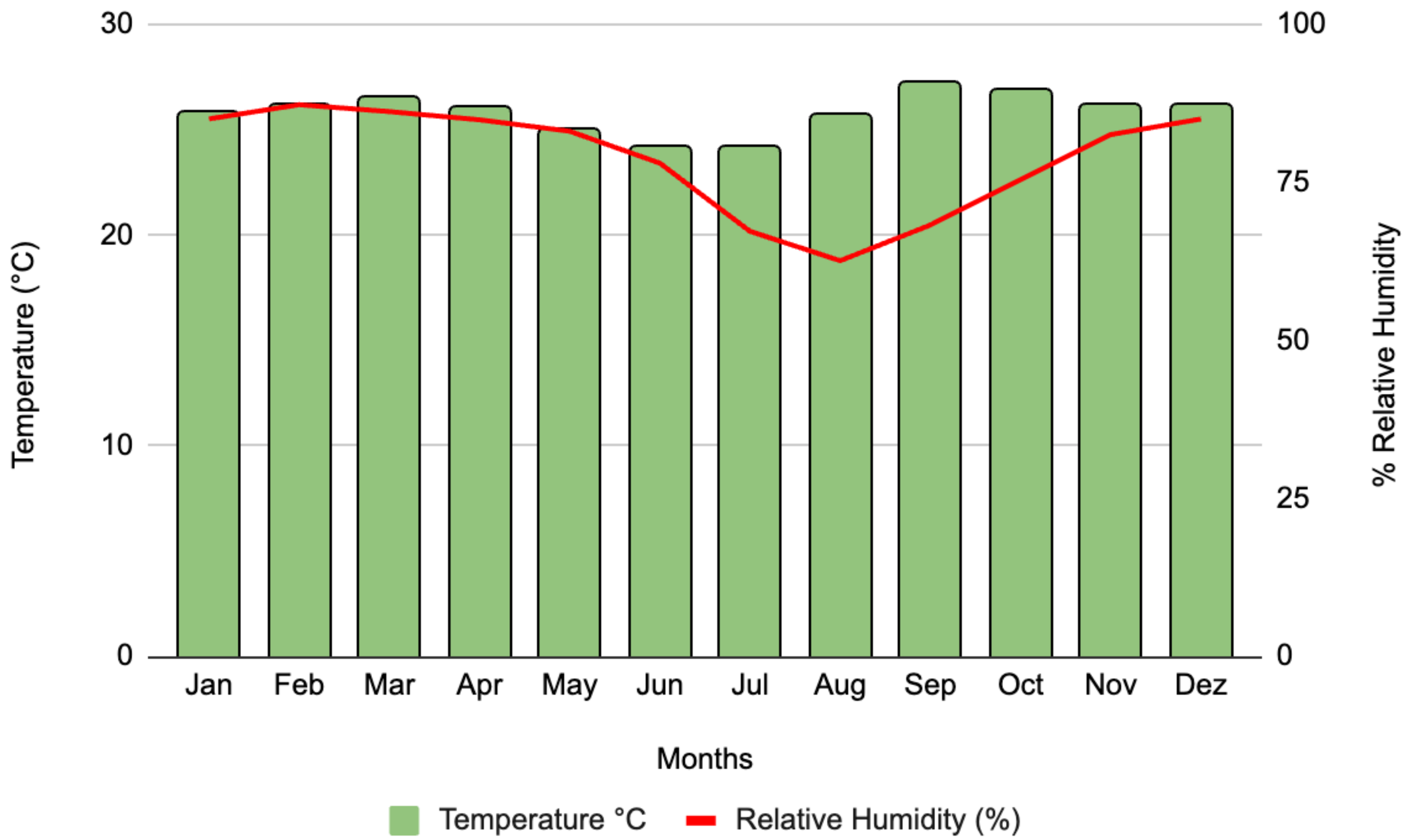

Supplementary Figure 1. Average monthly temperature (°C) and relative humidity (%) in the state of Mato Grosso, Brazil, based on data from 2013 to 2024.
